# The Pseudorabies Virus DNA Polymerase Accessory Subunit UL42 Directs Nuclear Transport of the Holoenzyme

**DOI:** 10.3389/fmicb.2016.00124

**Published:** 2016-02-15

**Authors:** Yi-Ping Wang, Wen-Juan Du, Li-Ping Huang, Yan-Wu Wei, Hong-Li Wu, Li Feng, Chang-Ming Liu

**Affiliations:** Division of Swine Infectious Diseases, State Key Laboratory of Veterinary Biotechnology, Harbin Veterinary Research Institute, Chinese Academy of Agricultural SciencesHarbin, China

**Keywords:** pseudorabies virus, DNA polymerase, accessory subunit, UL42, nuclear transport

## Abstract

Pseudorabies virus (PRV) DNA replication occurs in the nuclei of infected cells and requires the viral DNA polymerase. The PRV DNA polymerase comprises a catalytic subunit, UL30, and an accessory subunit, UL42, that confers processivity to the enzyme. Its nuclear localization is a prerequisite for its enzymatic function in the initiation of viral DNA replication. However, the mechanisms by which the PRV DNA polymerase holoenzyme enters the nucleus have not been determined. In this study, we characterized the nuclear import pathways of the PRV DNA polymerase catalytic and accessory subunits. Immunofluorescence analysis showed that UL42 localizes independently in the nucleus, whereas UL30 alone predominantly localizes in the cytoplasm. Intriguingly, the localization of UL30 was completely shifted to the nucleus when it was coexpressed with UL42, demonstrating that nuclear transport of UL30 occurs in an UL42-dependent manner. Deletion analysis and site-directed mutagenesis of the two proteins showed that UL42 contains a functional and transferable bipartite nuclear localization signal (NLS) at amino acids 354–370 and that K^354^, R^355^, and K^367^ are important for the NLS function, whereas UL30 has no NLS. Coimmunoprecipitation assays verified that UL42 interacts with importins α3 and α4 through its NLS. *In vitro* nuclear import assays demonstrated that nuclear accumulation of UL42 is a temperature- and energy-dependent process and requires both importins α and β, confirming that UL42 utilizes the importin α/β-mediated pathway for nuclear entry. In an UL42 NLS-null mutant, the UL42/UL30 heterodimer was completely confined to the cytoplasm when UL42 was coexpressed with UL30, indicating that UL30 utilizes the NLS function of UL42 for its translocation into the nucleus. Collectively, these findings suggest that UL42 contains an importin α/β-mediated bipartite NLS that transports the viral DNA polymerase holoenzyme into the nucleus in an *in vitro* expression system.

## Introduction

Pseudorabies virus (PRV), also known as *Suid herpesvirus 1* or Aujeszky's disease virus, is an economically important etiological agent of swine diseases, causing devastating diseases worldwide (Pomeranz et al., 2005; An et al., 2013; Yu et al., 2014). It is a member of the genus *Varicellovirus* in the subfamily *Alphaherpesvirinae* of the family *Herpesviridae* (Mettenleiter, 2000; Klupp et al., 2004). PRV is a double-stranded DNA virus and its genome is up to 143 kb long, encoding more than 70 different functional proteins (Pomeranz et al., 2005). PRV has a broad host range, infecting most mammals, and can be cultured in a wide variety of cell lines, including a porcine kidney cell line (PK-15) and a human cervical cancer cell line (HeLa). The DNAs of herpesviruses are replicated in the nuclei of the infected cells, which requires a set of virally encoded enzymatic proteins (Wu et al., 1988; Anders and McCue, 1996). For example, in Herpes simplex virus 1 (HSV-1), an important human pathogen of the subfamily *Alphaherpesvirinae*, seven proteins are directly involved in viral DNA replication (Lehman and Boehmer, 1999). All these proteins are conserved in PRV and are thought to function similarly (Pomeranz et al., 2005). Among of these proteins, the most important is the viral DNA polymerase, composed of a catalytic subunit UL30 with inherent DNA polymerase activity and an accessory subunit UL42, also called the “processivity factor,” that confers processivity on the holoenzyme (Purifoy et al., 1977; Gottlieb et al., 1990; Berthomme et al., 1995). The DNA polymerases of the herpesviruses are essential for their DNA replication (Wu et al., 1988; Lehman and Boehmer, 1999). To initiate viral DNA replication, the DNA polymerase must be transported into the nucleus after its synthesis in the cytoplasm, a prerequisite for its function. To date, the pathways by which the DNA polymerase catalytic and accessory subunits of several herpesviruses gain access to the nucleus have been clarified, such as HSV-1 UL30 and UL42 (Alvisi et al., 2007, 2008), Human cytomegalovirus (HCMV) UL54 and UL44 (Alvisi et al., 2005, 2006), Epstein-Barr virus (EBV) BALF5 and BMRF1 (Zhang et al., 1999; Kawashima et al., 2013), and Kaposi's sarcoma-associated herpesvirus (KSHV) Pol-8 and PF-8 (Chen et al., 2005). Intriguingly, it was demonstrated that nuclear transport of HSV-1 UL30 and EBV BALF5 was strongly inhibited by the inhibitors of heat shock protein 90 (Hsp90), leading to decreased viral yields and viral DNA synthesis, indicating that nuclear translocation of UL30 and BALF5 depends on molecular chaperone Hsp90 (Burch and Weller, 2005; Kawashima et al., 2013). However, the mechanisms of nuclear import of the PRV DNA polymerase catalytic subunit UL30 and accessory subunit UL42 remain unclear.

The trafficking of proteins between the cytoplasm and the nucleus occurs through large, proteinaceous structures called “nuclear pore complexes” (NPC), which are composed of approximately 30 proteins, collectively known as “nucleoporins” (Stoffler et al., 1999; Allen et al., 2000; Fahrenkrog and Aebi, 2003; Alber et al., 2007). The NPC spans the nuclear membrane and generates a pore channel with a diameter of 9 nm, which allows the passive diffusion of ions and small proteins (less than 60–70 kDa), but restricts the passage of larger molecules carrying specific targeting signals (Cardarelli et al., 2007; Lange et al., 2007). Therefore, the nucleocytoplasmic transport of larger proteins is mediated by an active mechanism governed by specific transport receptors and the corresponding *cis*-acting transport signals, called “nuclear localization signals” (NLSs) and “nuclear export signals” (Görlich and Kutay, 1999; Lischka et al., 2003). The NLSs are generally grouped into classical and nonconventional categories. The classical NLSs (cNLSs) are most common, and consist of clusters of basic amino acids in close proximity within the protein sequences. The cNLSs are categorized into three patterns: pat4, pat7, or bipartite. The pat4 motif is composed of four consecutive basic amino acids [(K/R)_4_], whereas the pat7 motif is composed of a proline separated by an interval of 1–3 amino acids from four residues, three of which are basic [P(X)_1−3_(K/R)_3−4_, where X represents any amino acid] (Macara, 2001; Boisvert et al., 2014). Compared with these monopartite NLS motifs, typified by the Simian virus 40 (SV40) large T-antigen (TAg) NLS (^126^PKKKRKV^132^) (Kalderon et al., 1984a,b), the bipartite motif consists of two stretches of basic residues separated by a 10–12 amino acid linker [KR(X)_10−12_K(K/R)X(K/R)], typified by the *Xenopus laevis* nucleoplasmin NLS (^155^KRPAATKKAGQAKKKK^170^) (Dingwall et al., 1988).

Proteins containing cNLSs are transported into the nucleus by the classical nuclear import pathway, involving nuclear transport receptors importin α (also known as “karyopherin α”) and importin β (also called “karyopherin β”) (Marfori et al., 2011, 2012). Seven different isoforms of importin α (α1, α3, α4, α5, α6, α7, and α8) have been identified in mammalian cells, which fall into three subfamilies α-P (α1 and α8), α-Q (α3 and α4), and α-S (α5, α6, and α7), differing in their cargo specificity and affinity (Hogarth et al., 2006; Tejomurtula et al., 2009; Kelley et al., 2010). As the adaptor molecule, importin α mediates the interaction between the cNLS-containing cargo proteins and importin β (Adam and Adam, 1994; Görlich et al., 1994; Radu et al., 1995). Importin α binds to the cNLS-containing cargoes *via* its major and minor NLS-binding sites and binds to importin β *via* its N-terminal importin-β-binding (IBB) domain (Marfori et al., 2012, 2011). The IBB domain has been shown to interact with the major and minor NLS-binding sites, thus autoinhibiting importin α from NLS binding (Kobe, 1999; Fontes et al., 2003b). The cNLS-dependent nuclear import pathway can be viewed as a two-step process (Bian et al., 2007). The first step is the assembly of the heterotrimeric complex composed of the importin α/β–cNLS-containing-protein complex, followed by the binding of importin β to the NPC. The second step is the translocation of the heterotrimer into the nucleus, where importin β is released from the complex after binding to nuclear RanGTP, causing the dissociation of the cNLS-bearing cargo from the transport receptors and the subsequent recycling of importins α and β back to the cytoplasm for another round of importation (Görlich and Kutay, 1999; Macara, 2001). The directionality of nucleocytoplasmic transport is imparted by the small GTPase protein Ran, which is dependent on the gradient of RanGTP (enriched in the nucleus) to RanGDP (abundant in the cytoplasm) (Kalab et al., 2002; Smith et al., 2002). Therefore, the import receptors bind to the cargo protein in the cytoplasm in the absence of RanGTP and release it in the nucleus after RanGTP binds to importin β (Moroianu et al., 1996; Lange et al., 2007).

In this study, we demonstrated that nuclear transport of the PRV DNA polymerase catalytic subunit UL30 is dependent on the accessory subunit UL42, whereas UL42 is transported into the nucleus through the classical importin α/β-mediated nuclear import pathway. It has been shown that UL42 contains a functional and transferable bipartite NLS that not only mediates nuclear localization of free UL42 but also directs nuclear transport of the UL42/UL30 heterodimer. Our overall results indicate that the bipartite NLS in UL42 is a key regulatory motif directing nuclear import of the PRV DNA polymerase holoenzyme.

## Materials and methods

### Cells and viruses

PK-15, HEK293T, and HeLa cells were maintained in Dulbecco's modified Eagle's medium (DMEM; Gibco-BRL, Grand Island, NY) supplemented with 10% fetal bovine serum (FBS), 100 μg/ml streptomycin, and 100 IU/ml penicillin. HEK293T cells were used in the coimmunoprecipitation (co-IP) assays and subsequent western blotting analysis, whereas HeLa cells were used in the subcellular localization experiments. The PRV-JF viral strain was previously isolated from a pig farm by our laboratory (Zhang et al., 2008), propagated in PK-15 cells, and used for the amplification of the UL42 and UL30 genes.

### Plasmid construction

The full-length UL42 (GenBank accession: KP279683) and UL30 genes (GenBank accession: KP279684) were amplified from the DNA extracted from PRV-JF-strain-infected PK-15 cells and cloned into the vectors pCMV-N-Flag (Beyotime, Shanghai, China) and pCAGGS-HA (Addgene, Cambridge, MA), respectively, generating two recombinant plasmids pCMV-Flag–UL42 and pCAGGS-HA–UL30, respectively. Various plasmids expressing enhanced green fluorescent protein (EGFP)–UL42 fusion proteins were generated by inserting the corresponding full-length and truncated UL42 fragments into the *Hin*dIII and *Eco*RI sites in the pEGFP-C1 vector (Clontech, Palo Alto, CA), generating recombinant expression plasmids pEGFP–UL42(2–384), pEGFP–UL42(2–366), pEGFP–UL42(367–384), pEGFP–UL42(2–363), pEGFP–UL42(364–384), pEGFP–UL42(2–370), pEGFP–UL42(371–384), pEGFP–UL42(2–353), and pEGFP–UL42(354–384). For the positive control, the SV40 TAg-NLS (PKKKRKV) was cloned into the *Hin*dIII and *Eco*RI sites of pEGFP-C1 to generate pEGFP–SV40NLS. To generate the NLS-null recombinant plasmid pCMV-Flag–UL42ΔNLS, the NLS domain was deleted from pCMV-Flag–UL42 using a specific forward primer comprising nucleotides 1030–1059 and 1111–1140 of the UL42 opening reading frame and its complementary sequence, according to the QuickChange Site-Directed Mutagenesis Kit instructions (Stratagene, La Jolla, CA).

To create pEGFP–β-Gal, the full-length β-galactosidase (β-Gal) gene was amplified from *Escherichia coli* (*E. coli*) and inserted into the *Pst*I and *Bam*HI sites of pEGFP-C1. The SV40 TAg-NLS, predicted UL42 NLS (pat4, pat7, or bipartite), and the mutated UL42 bipartite NLS [with K354A, R355A, KR(354–355)AA, R360A, K367A, R368A, or R370A substitution] were cloned into the *Hin*dIII and *Pst*I sites of pEGFP–β-Gal, generating the following recombinant plasmids, pEGFP–SV40NLS–β-Gal, pEGFP–Pat4–β-Gal, pEGFP–Pat7–β-Gal, pEGFP–Bipartite–β-Gal, pEGFP–K354A–β-Gal, pEGFP–R355A–β-Gal, pEGFP–(354–355)A–β-Gal, pEGFP–R360A–β-Gal, pEGFP–K367A–β-Gal, pEGFP–R368A–β-Gal, and pEGFP–R370A–β-Gal, respectively.

To generate plasmids expressing hemagglutinin (HA)-tagged importins α and β, the importins α1, α3, α4, α5, α6, α7, and α8 genes lacking the N-terminal IBB domains were amplified from the cDNA from HeLa cells and cloned into the *Eco*RI and *Xho*I sites of pCAGGS-HA. Similarly, the full-length importin β gene was amplified and cloned into the *Cla*I and *Sph*I sites of pCAGGS-HA. To generate pCAGGS-His–Impα4, pCAGGS-HA was first reconstructed to pCAGGS–His (a kind gift from Dr. Jianfei Chen at our institute) by replacing the HA tag with a His tag, and then the full-length importin α4 gene was amplified and cloned into the *Cla*I and *Sph*I sites of pCAGGS–His.

All the plasmid constructs described above were confirmed with DNA sequencing and no unwanted mutation, deletion, or insertion was detected. The sequence information for all the primers used in this study is summarized in Table 1.

**Table 1:** **Primers used in this study**.

**Name^a^**	**Sequence (5′–3′)^e^**	**Constructs**
UL42–HindIII-F	CCCAAGCTTTCGCTGTTCGACGACGGCCTCGAGGACCTG	pCMV-Flag–UL42
UL42–EcoRI-R^b^	CCGGAATTCTTAGAATAAATCTCCGTAGGCGTGGCCCCC	
UL30–EcoRI-F	CCGGAATTCGCGGCGCGGCAGGGCAGCTACGTGACGCGC	pCAGGS-HA–UL30
UL30–SphI-R	ACATGCATGCTCAGCTTCGACGGGGAGCTGCTGTTGGAGT	
UL42(2–384)-F^c^	CCCAAGCTTCTTCGCTGTTCGACGACGGCCTCGAGGACCTG	pEGFP–UL42(2–384)
UL42(2–370)-R	CCGGAATTCTTACCGCGGGCGCTTGGCGATGGGCGTGTA	pEGFP–UL42(2–370)
UL42(2–366)-R	CCGGAATTCTTAGGCGATGGGCGTGTACATGCGCGGGGCGGCGGGCC	pEGFP–UL42(2–366)
UL42(2–363)-R	CCGGAATTCTTACGTGTACATGCGCGGGGCGGCGGGCCGCTTGT	pEGFP–UL42(2–363)
UL42(2–353)-R	CCGGAATTCTTAGTCCCCCGCGGCGGGGGCGGCGGAGGA	pEGFP–UL42(2–353)
UL42(354–384)-F	CCCAAGCTTCTAAGCGGCCCGCCGCCCCGCGCATGTACACG	pEGFP–UL42(354–384)
UL42(364–384)-F	AGCTTCTCCCATCGCCAAGCGCCCGCGGACCGCCTCGGGGGAAGGGGGCCACGCCTAC GGAGATTTATTCTAAG	pEGFP–UL42(364–384)^f^
UL42(364–384)-R	AATTCTTAGAATAAATCTCCGTAGGCGTGGCCCCCTTCCCCCGAGGCGGTCCGCGGG CGCTTGGCGATGGGAGA	
UL42(367–384)-F	AGCTTCTAAGCGCCCGCGGACCGCCTCGGGGGAAGGGGGCCACGCCTACGGAGAT TTATTCTAAG	pEGFP–UL42(367–384)^f^
UL42(367–384)-R	AATTCTTAGAATAAATCTCCGTAGGCGTGGCCCCCTTCCCCCGAGGCGGTCCGCGG GCGCTTAGA	
UL42(371–384)-F	AGCTTCTACCGCCTCGGGGGAAGGGGGCCACGCCTACGGAGATTTATTCTAAG	pEGFP–UL42(371–384)^f^
UL42(371–384)-R	AATTCTTAGAATAAATCTCCGTAGGCGTGGCCCCCTTCCCCCGAGGCGGTAGA	
SV40NLS-F	AGCTTCTCCAAAAAAGAAGAGAAAGGTAG	pEGFP–SV40NLS^f^
SV40NLS-R	AATTCTACCTTTCTCTTCTTTTTTGGAGA	
UL42ΔNLS-F	GCGTCCTCCGCCGCCCCAGCAGCAGGAGATACCGCCTCGGGGGAAGGGGGCCACG CCTAC	pCMV-Flag–UL42ΔNLS
UL30(539–541)m-F^d^	CCCCCGCCGCGGGGGACGCAGCACCAGCAGCCCCGCGCATGTACA	pCAGGS-HA–UL30^(539−541)A^
β-Gal–PstI-F	AAAACTGCAGTCACCATGATTACGGATTCACTGGCCGTCGTA	pEGFP–β-Gal
β-Gal–BamHI-R	CGCGGATCCTTATTTTTGACACCAGACCAACTGGTAATG	
SV40NLS–β-Gal-F	AGCTTCTCCAAAAAAGAAGAGAAAGGTATCTGCA	pEGFP–SV40NLS–β-Gal^g^
SV40NLS–β-Gal-R	GATACCTTTCTCTTCTTTTTTGGAGA	
Pat4–β-Gal-F	AGCTTCTAAGCGCCCGCGGTCTGCA	pEGFP–Pat4–β-Gal^g^
Pat4–β-Gal-R	GACCGCGGGCGCTTAGA	
Pat7–β-Gal-F	AGCTTCTCCCATCGCCAAGCGCCCGCGGTCTGCA	pEGFP–Pat7–β-Gal^g^
Pat7–β-Gal-R	GACCGCGGGCGCTTGGCGATGGGAGA	
Bipartite–β-Gal-F	AGCTTCTAAGCGGCCCGCCGCCCCGCGCATGTACACGCCCATCGCCAAGCGCCCG CGGTCTGCA	pEGFP–Bipartite–β-Gal^g^
Bipartite–β-Gal-R	GACCGCGGGCGCTTGGCGATGGGCGTGTACATGCGCGGGGCGGCGGGCCGCTTAGA	
K354A–β-Gal-F	AGCTTCGGCACGGCCCGCCGCCCCGCGCATGTACACGCCCATCGCCAAGCGCCCGCG GTCTGCA	pEGFP–K354A–β-Gal^g^
K354A–β-Gal-R	GACCGCGGGCGCTTGGCGATGGGCGTGTACATGCGCGGGGCGGCGGGCCGTGCAGA	
R355A–β-Gal-F	AGCTTCGAAGGCACCCGCCGCCCCGCGCATGTACACGCCCATCGCCAAGCGCCCGCG GTCTGCA	pEGFP–R355A–β-Gal^g^
R355A–β-Gal-R	GACCGCGGGCGCTTGGCGATGGGCGTGTACATGCGCGGGGCGGCGGGTGCCTTAGA	
(354–355)A–β-Gal-F	AGCTTCGGCAGCACCCGCCGCCCCGCGCATGTACACGCCCATCGCCAAGCGCCCGCG GTCTGCA	pEGFP–(354–355)A–β-Gal^g^
(354–355)A–β-Gal-R	GACCGCGGGCGCTTGGCGATGGGCGTGTACATGCGCGGGGCGGCGGGTGCTGCAGA	
R360A–β-Gal-F	AGCTTCTAAGCGGCCCGCCGCCCCGGCAATGTACACGCCCATCGCCAAGCGCCCGCG GTCTGCA	pEGFP–R360A–β-Gal^g^
R360A–β-Gal-R	GACCGCGGGCGCTTGGCGATGGGCGTGTACATTGCCGGGGCGGCGGGCCGCTTAGA	
K367A–β-Gal-F	AGCTTCTAAGCGGCCCGCCGCCCCGCGCATGTACACGCCCATCGCCGCACGCCCG CGGTCTGCA	pEGFP–K367A–β-Gal^g^
K367A–β-Gal-R	GACCGCGGGCGTGCGGCGATGGGCGTGTACATGCGCGGGGCGGCGGGCCGCTTAGA	
R368A–β-Gal-F	AGCTTCTAAGCGGCCCGCCGCCCCGCGCATGTACACGCCCATCGCCAAGGCACCGCG GTCTGCA	pEGFP–R368A–β-Gal^g^
R368A–β-Gal-R	GACCGCGGTGCCTTGGCGATGGGCGTGTACATGCGCGGGGCGGCGGGCCGCTTAGA	
R370A–β-Gal-F	AGCTTCTAAGCGGCCCGCCGCCCCGCGCATGTACACGCCCATCGCCAAGCGCCCGGC ATCTGCA	pEGFP–R370A–β-Gal^g^
R370A–β-Gal-R	GATGCCGGGCGCTTGGCGATGGGCGTGTACATGCGCGGGGCGGCGGGCCGCTTAGA	
Impα1–EcoRI-F	CCGGAATTCGCCAGGAAACTACTTTCCAGAGAAAAACAG	pCAGGS-HA–Impα1
Impα1–XhoI-R	CCGCTCGAGCTAAAAGTTAAAGGTCCCAGGAGCCCCATC	
Impα3–EcoRI-F	CCGGAATTCGCTAGGAAGCTTTTGTCCAGTGATCGAAAT	pCAGGS-HA–Impα3
Impα3–XhoI-R	CCGCTCGAGCTAAAACTGGAACCCTTCTGTTGGTACATT	
Impα4–EcoRI-F	CCGGAATTCGCAAGAAAACTGTTATCCAGTGACAGAAAT	pCAGGS-HA–Impα4
Impα4–XhoI-R	CCGCTCGAGTTAAAAATTAAATTCTTTTGTTTGAAGGTT	
Impα5–EcoRI-F	CCGGAATTCGAGCAACAGCTTTCAGCAACACAGAAATTC	pCAGGS-HA–Impα5
Impα5–XhoI-R	CCGCTCGAGTCAAAGCTGGAAACCTTCCATAGGAGCCTC	
Impα6–EcoRI-F	CCGGAATTCGATCAACAGCTAACAGCAACACAGAAATTT	pCAGGS-HA–Impα6
Impα6–XhoI-R	CCGCTCGAGTTAAAGTTGAAATCCATCCATTGGTGCTTC	
Impα7–EcoRI-F	CCGGAATTCTTCCGGAAACTGCTCTCCAAAGAGCCTAGT	pCAGGS-HA–Impα7
Impα7–XhoI-R	CCGCTCGAGTTATAGCTGGAAGCCCTCCATGGGGGCCTC	
Impα8–EcoRI-F	CCGGAATTCGCCAGGAAAATGCTATCCCAGGAAAAGAAC	pCAGGS-HA–Impα8
Impα8–XhoI-R	CCGCTCGAGCTATTTTTTTGCTAAGCATTCATAATCTAT	
Impβ–ClaI-F	CCATCGATGAGCTGATCACCATTCTCGAGAAGACCGTG	pCAGGS-HA–Impβ
Impβ–SphI-R	ACATGCATGCTCAAGCTTGGTTCTTCAGTTTCCTCAGTTC	
Impα4–ClaI-F	CCATCGATGCCGAGAACCCCAGCTTGGAGAACCACCGC	pCAGGS-His–Impα4
Impα4–SphI-R	ACATGCATGCTTAAAAATTAAATTCTTTTGTTTGAAGGTT	

a Impα and Impβ are the abbreviations of importin α and importin β, respectively.

b The sequences of the primers UL42(2–384)-R and UL42(354–384)-R are consistent with the sequence of the primer UL42-EcoRI-R.

c The sequences of the primers UL42(2–370)-F, UL42(2–366)-F, UL42(2–363)-F, and UL42(2–353)-F are consistent with the sequence of the primer UL42(2–384)-F.

d UL30(539–541)m-F and its corresponding complementary reverse primer were used for the construction of pCAGGS-HA–UL30^(539-541)A^, according to the QuickChange Site-Directed Mutagenesis Kit instructions, in which ^539^KRR^541^ is substituted with three alanine residues.

e The underlined sequences represent the restriction sites introduced for convenient cloning. Of the primers [UL42ΔNLS-F and UL30(539–541)m-F] designed for site-directed mutagenesis, only the forward primers are shown, but the corresponding complementary reverse primers were also used.

f Recombinant plasmids pEGFP–UL42(364–384), pEGFP–UL42(367–384), pEGFP–UL42(371–384), and pEGFP–SV40NLS were constructed by inserting the corresponding annealed primer pairs into the HindIII and EcoRI sites of pEGFP-C1.

g Recombinant plasmids pEGFP–SV40NLS–β-Gal, pEGFP–Pat4–β-Gal, pEGFP–Pat7–β-Gal, pEGFP–Bipartite–β-Gal, pEGFP–K354A–β-Gal, pEGFP–R355A–β-Gal, pEGFP–(354–355)A–β-Gal, pEGFP–R360A–β-Gal, pEGFP–K367A–β-Gal, pEGFP–R368A–β-Gal, and pEGFP–R370A–β-Gal were constructed by inserting the corresponding annealed primer pairs into the HindIII and PstI sites of pEGFP–β-Gal.

### Transfection of EGFP-expressing fusion constructs

HeLa cells were propagated in DMEM medium containing 10% FBS at 37°C under 5% CO_2_ in a humidified incubator. The cells were plated in 20 mm glass-bottom cell culture dishes (Nest, Wuxi, China) 1 day before transfection and grown to 50% confluence by the following day. The cells were transfected with recombinant plasmids expressing EGFP fused to full-length UL42 or to truncated or mutated UL42 derivatives using Attractene Transfection Reagent (Qiagen, Dusseldorf, Germany), according to the manufacturer's recommendations. At 24 h posttransfection, the cells were fixed with 4% paraformaldehyde in phosphate-buffered saline (PBS, pH 7.4) for 30 min, and the DNA was stained with Hoechst 33258 (10 μg/ml; Sigma, St. Louis, MO). The subcellular localization of the different EGFP–UL42 fusion proteins was visualized with a Leica SP2 confocal system (Leica Microsystems, Wetzlar, Germany). The intracellular distribution of the various fusion proteins was analyzed semiquantitatively, based on the EGFP fluorescent signals, and the results were expressed as the percentages of cells in each of the five categories of protein localization: (1) exclusive nuclear localization (N); (2) more nuclear than cytoplasmic accumulation (N > C); (3) diffuse throughout the cells (N = C); (4) more cytoplasmic than nuclear accumulation (C > N); (5) strict cytoplasmic localization (C). For each fusion protein, 100 EGFP-expressing cells were randomly selected and categorized. Each transfection experiment was performed in triplicate. The results were analyzed using one-way repeated measurement ANOVA followed by least significance difference (LSD) in the SAS system for Windows version 8.1 (SAS Institute Inc., Cary, NC). The significance level (α) was set at 0.05, and *P* < 0.05 was considered to indicate a statistically significant difference.

### Immunofluorescence and confocal microscopy

HeLa cells were separately transfected or cotransfected with pCMV-Flag–UL42 or pCMV-Flag–UL42ΔNLS and pCAGGS-HA–UL30 in 20 mm glass-bottom cell culture dishes (Nest) with Attractene Transfection Reagent (Qiagen). After incubation for 24 h, the cells were fixed with 4% paraformaldehyde in PBS for 30 min and permeabilized with 0.2% Triton X-100 in PBS for 10 min. The cells were then incubated at 37°C for 1 h with mouse anti-Flag M2 monoclonal antibody [MAb, diluted 1:500 in PBS containing 1% bovine serum albumin (BSA); Sigma] and/or rabbit anti-HA polyclonal antibody (PcAb, diluted 1:50 in PBS containing 1% BSA; Sigma) as the primary antibodies. After the cells were washed three times, they were incubated at 37°C for 1 h with secondary antibodies, including goat anti-mouse and/or anti-rabbit IgG antibodies conjugated with DyLight™ 488 or 594 (diluted 1:500 in PBS containing 1% BSA; Pierce, Rockford, IL). Finally, DNA was stained with Hoechst 33258 (Sigma) for 10 min at room temperature and the fluorescent signals were visualized with a Leica SP2 confocal system (Leica Microsystems).

### Co-IP and western blotting

HEK293T cells were transfected, as described above, with the indicated recombinant plasmids in the corresponding figure legends. The cells were harvested 24 h after transfection, washed twice with cold PBS, and lysed with Triton X-100 buffer [20 mM Tris (pH 7.5), 150 mM NaCl, 1% Triton X-100, and 0.5 mM EDTA] supplemented with 1 mM phenylmethylsulfonyl fluoride (PMSF) at 4°C for 30 min. The cell lysates were clarified by centrifugation and subjected to co-IP assays. IP was performed with protein A/G Plus–Agarose Immunoprecipitation Reagent (Santa Cruz Biotechnology, Dallas, TX), according to the manufacturer's instructions. Briefly, the cell lysates were first precleared with 20 μl of protein A/G agarose for 2 h at 4°C. After centrifugation, the supernatants were subjected to IP with a mouse anti-Flag M2 MAb (diluted 1:50; Sigma) or mouse anti-HA MAb (1:100; Sigma) and 40 μl of protein A/G agarose, with incubation overnight at 4°C. The beads were rinsed with PBS, pelleted in electrophoresis sample buffer, and boiled for 3 min. The protein samples were separated with 12% SDS-PAGE and then transferred onto nitrocellulose membranes. Western blotting was performed with a mouse anti-Flag M2 MAb (diluted 1:2000; Sigma), mouse anti-HA MAb (diluted 1:20000; Sigma), rabbit anti-importin α3 PcAb (diluted 1:500; Proteintech, Chicago, IL), rabbit anti-importin α4 PcAb (diluted 1:2000; Pierce), mouse anti-importin β MAb (diluted 1:2000; Sigma), or mouse anti-β-actin MAb (diluted 1:2000; Pierce) as the primary antibody, followed by the IRDye 800CW Goat anti-Mouse IgG (H+L) antibody (diluted 1:10000; Li-Cor Biosciences, Lincoln, NE) and the IRDye 680RD goat anti-rabbit IgG (H+L) (diluted 1:10000; Li-Cor Biosciences) as the secondary antibodies. The protein blots were scanned with an Odyssey Infrared Imaging System (Li-Cor Biosciences).

### Protein expression and purification

Eight 75 cm^2^ flasks of HEK293T cells were transfected with pCMV-Flag–UL42, pCMV-Flag–UL42ΔNLS, or pCAGGS-His–Impα4, as described above, with 10 μg of plasmid in each flask. The cells were collected 24 h after transfection, washed twice with PBS, and lysed with Triton X-100 buffer containing 1 mM PMSF. After clarification by centrifugation, the cell lysates were subjected directly to the protein purification procedure. Flag–UL42 and Flag–UL42ΔNLS were purified with Anti-DYKDDDDK G1 Affinity Resin (GenScript, Piscataway, NJ), according to the manufacturer's recommendations. The purified proteins were eluted with 3 M NaCl buffer (pH 7.4), and subsequently desalted with PD-10 Desalting Columns (GE Healthcare, Uppsala, Sweden), with transport buffer (TB; described in the section “Nuclear import assays”) as the elution buffer. His–Impα4 was purified with Anti-His Affinity Resin (GenScript), according to the manufacturer's instructions. The purified His–Impα4 fusion protein was eluted with alkaline elution buffer [0.1 M Tris (pH 12.0), 0.5 M NaCl], and 50 μl of 1 M HCl was added per milliliter of eluate. The eluate was then subjected to buffer exchange with TB using PD-10 Desalting Columns (GE Healthcare). All three purified proteins were stored at –80°C until analysis.

### Nuclear import assays

*In vitro* nuclear import assays using digitonin-permeabilized HeLa cells were performed as previously described by Maertens et al. (2004), with minor modifications. Briefly, HeLa cells were seeded in 20 mm glass-bottom cell culture dishes (Nest) until they reached 50–60% confluence. The cells were washed three times with ice-cold TB [20 mM HEPES (pH 7.3), 2 mM magnesium acetate, 110 mM potassium acetate, 2 mM dithiothreitol, 1 mM EGTA, 1 μg/ml each of aprotinin, leupeptin, and pepstatin, 1 mM PMSF] and permeabilized with 50 μg/ml digitonin in TB for 5 min at room temperature. After the cells were washed three times with ice-cold TB, they were incubated with TB alone or with 50 μg/ml wheat-germ agglutinin (WGA; Sigma) in TB for 15 min at 37°C. The endogenous cytosol was then removed by washing the cells five times with ice-cold TB. The cells were incubated for 30 min at 37°C with 100 μl of import reaction mixture containing 40 mg/ml rabbit reticulocyte lysate (RRL; Promega, Madison, WI), an ATP-regenerating system (1 mM ATP, 10 mM creatine phosphate, 20 units/ml creatine phosphokinase, 1 mM GTP; Sigma), and an import substrate of 200 μg/ml purified Flag–UL42 or Flag–UL42ΔNLS in TB. To examine the temperature dependence of importation, the cells were maintained on ice (0°C) during the incubation period of the import mixture. The ATP dependence of importation was tested by replacing ATP with 1 mM 5′-adenylimidodiphosphate (AMP–PNP; Sigma), and the GTP dependence was tested by adding 1 mM guanosine 5′-*O*-(3-thiotriphosphate) (GTPγS; Sigma) in place of GTP. Competition experiments were performed by the addition of 1 mM competitor peptides derived from the SV40 TAg-NLS (STPPKKKRKVED) or the heterogeneous nuclear ribonucleoprotein (hnRNP) A1-M9 domain (YNNQSSNFGPMK) (Lischka et al., 2003). For the reconstitution experiments, RRL was replaced with a Ran mixture composed of 3 μM Ran (Sigma) and 0.5 μM nuclear transport factor 2 (NTF2; Creative BioMart, Shirley, NY), with or without 1 μM purified importin α4 and/or 1 μM importin β (Sigma) in TB. To terminate the reaction, the cells were washed three times with TB. The cells were then fixed with 4% paraformaldehyde in PBS and subjected to immunofluorescence analyses performed as described above.

## Results

### Nuclear transport of the PRV DNA polymerase catalytic subunit UL30 is dependent on the accessory subunit UL42

**Figure 1:**
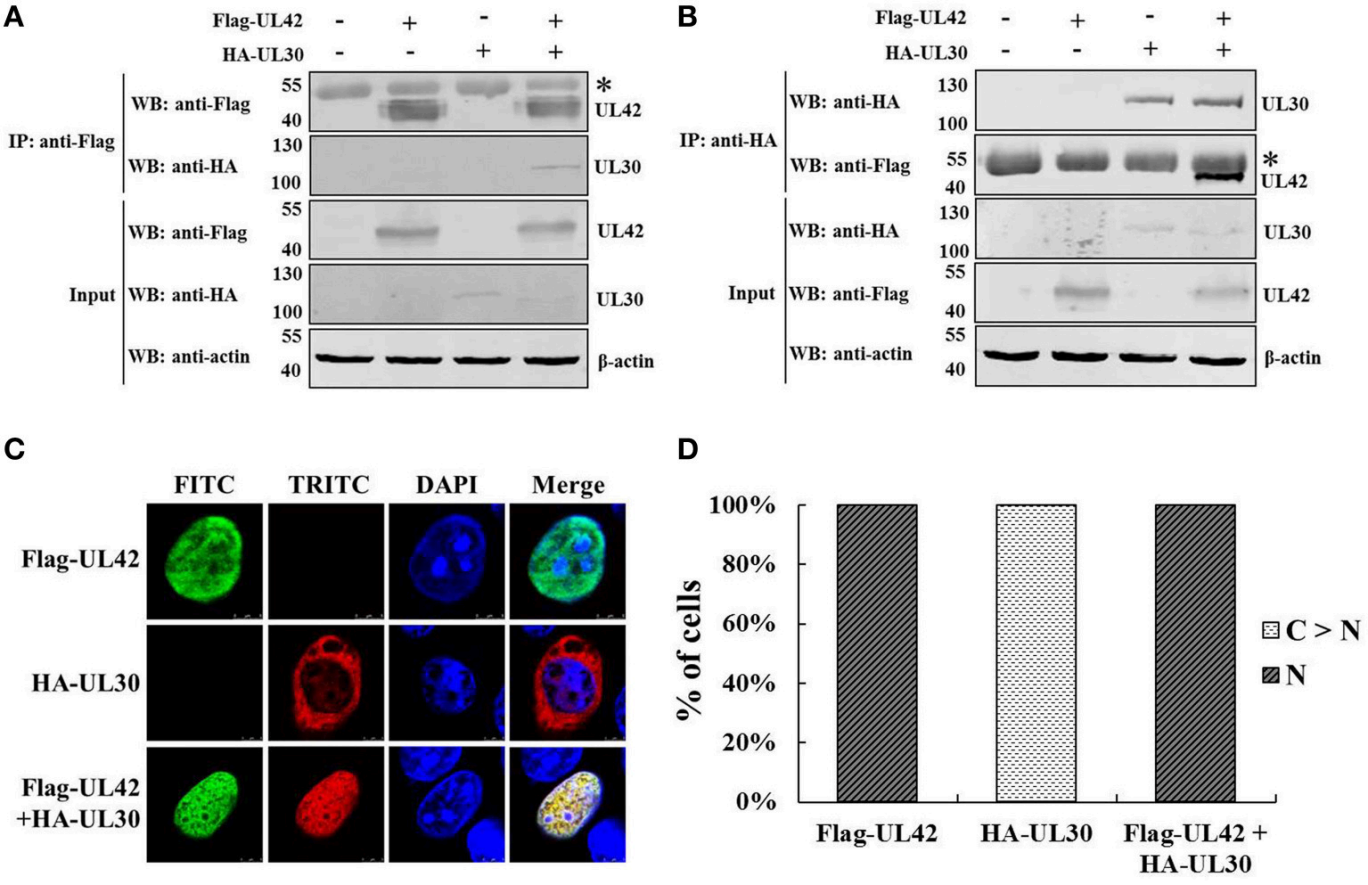
**Nuclear transport of the PRV DNA polymerase catalytic subunit UL30 requires the accessory subunit UL42. (A)** Co-IP of HEK293T cells transfected with pCMV-Flag–UL42 and/or pCAGGS-HA–UL30. IP was performed with an MAb recognizing the Flag tag, and the western blotting (WB) was probed with the antibodies indicated on the left. The cytoskeletal protein β-actin was used as the internal control. The asterisk indicates the heavy chain of IgG. The positions of the molecular mass markers (kDa) are indicated on the left. The WB results are representative of three or more independent experiments. **(B)** Reverse Co-IP of HEK293T cells transfected with pCMV-Flag–UL42 and/or pCAGGS-HA–UL30. IP was performed with an MAb recognizing the HA tag, and the WB was probed using the antibodies indicated on the left. The cytoskeletal protein β-actin was used as the internal control. The asterisk indicates the heavy chain of IgG. The positions of the molecular mass markers (kDa) are indicated on the left. The WB results are representative of three or more independent experiments. **(C)** Colocalization of UL42 and UL30 in the absence of other viral proteins. HeLa cells were transfected with pCMV-Flag–UL42 and/or pCAGGS-HA–UL30, fixed at 24 h posttransfection, and subjected to immunofluorescence analyses using antibodies directed against either the Flag tag (FITC, green) or the HA tag (TRITC, red) and the DNA was stained with Hoechst (DAPI, blue). The merged FITC, TRITC, and DAPI signals are shown. The images of each construct are representative of three independent transfection experiments. **(D)** To analyze the localization patterns of Flag–UL42 and HA–UL30 statistically, 100 positive cells expressing Flag–UL42 or HA–UL30 or coexpressing Flag–UL42 and HA–UL30 were scored from independent transfections in three repeated experiments and the relative percentages of the different subcellular localization categories were calculated. N, exclusively nuclear; C > N, more cytoplasmic than nuclear.

A previous study demonstrated that the PRV DNA polymerase accessory subunit UL42, but not the counterpart in HSV-1, stimulates the activity of the catalytic subunit UL30, implying a specific interaction between UL42 and UL30 (Berthomme et al., 1995). It is well known that the herpesviruses DNA polymerase catalytic subunits interact with their accessory subunits to form a heterodimeric complex, resulting in acquirement of enhanced processivity of the holoenzyme (Boehmer and Lehman, 1997). However, the association between the PRV DNA polymerase two subunits has not been confirmed experimentally. Here, we demonstrated the interaction between UL42 and UL30 using reciprocal co-IP assays. HA–UL30 were coimmunoprecipitated with Flag–UL42 only from the lysates of pCMV-Flag–UL42- and pCAGGS-HA–UL30-cotransfected cells, whether IP was performed with an anti-Flag (Figure 1A) or anti-HA MAb (Figure 1B), confirming that UL42 interacts with UL30 in the absence of other viral proteins, as do their counterparts in HSV-1 (Hernandez and Lehman, 1990; Digard et al., 1993; Gottlieb and Challberg, 1994).

The PRV DNA polymerase is critical for viral DNA replication. The nuclear localization of the DNA polymerase holoenzyme is a prerequisite for its function in the initiation of viral DNA replication. The subcellular localization of the PRV DNA polymerase two subunits was then investigated. As shown in Figures 1C,D, when transfected alone, UL42 was detected exclusively in the nucleus, like its counterpart in HSV-1 (Alvisi et al., 2008). Unexpectedly, UL30 alone was observed predominantly in the cytoplasm, with only faint staining in the nucleus (Figures 1C,D), in contrast to the nuclear localization of the corresponding UL30 of HSV-1 (Loregian et al., 2000; Alvisi et al., 2007). Intriguingly, UL42 and UL30 strictly colocalized in the nucleus in cotransfected cells (Figures 1C,D). These results clearly indicate that UL30 is unable to translocate independently to the nucleus and that its nuclear transport occurs in an UL42-dependent manner.

### UL42 contains a bipartite NLS, whereas UL30 has no NLS

**Figure 2:**
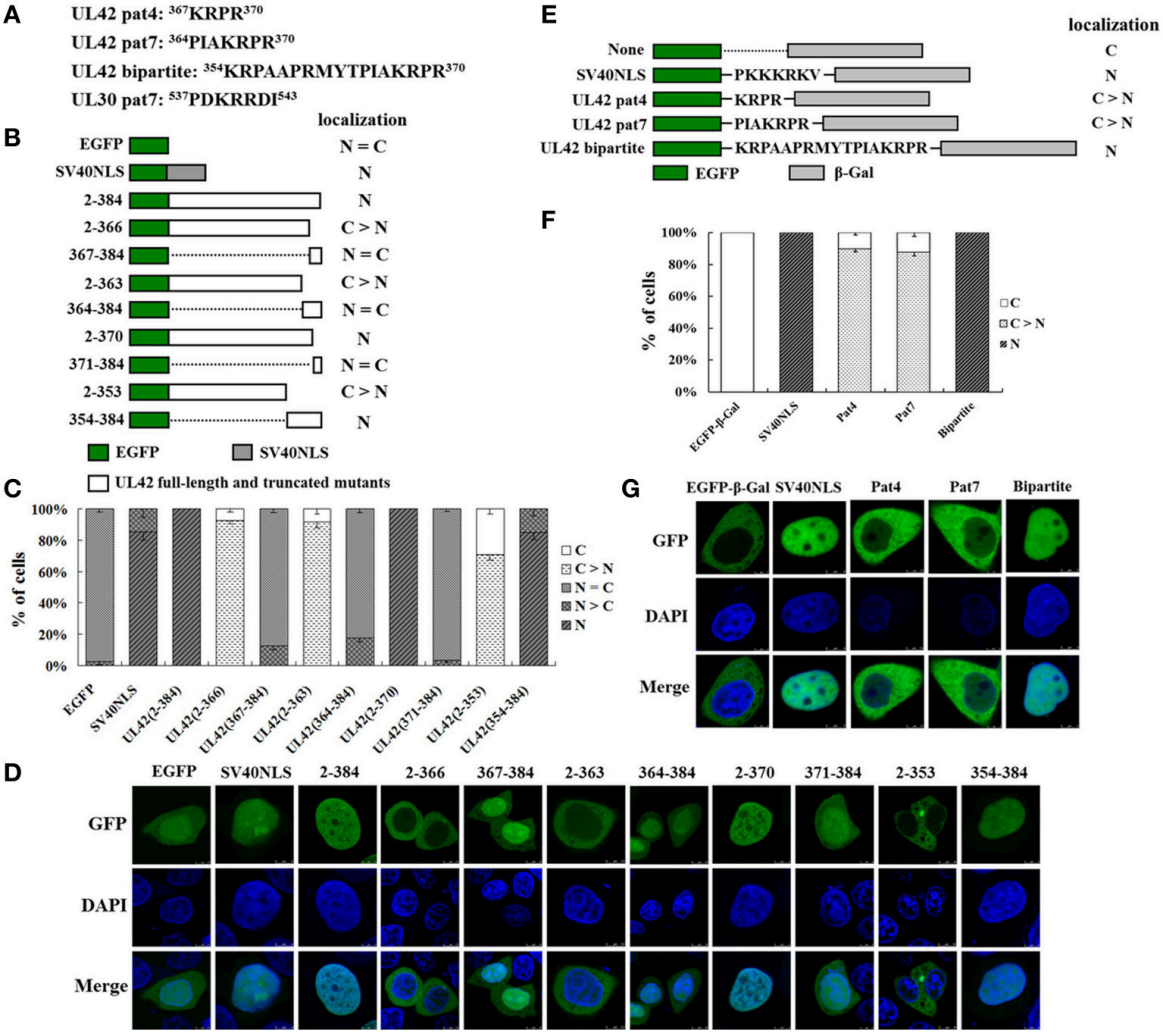
**UL42 contains a functional and transferable bipartite NLS that mediates its nuclear localization. (A)** The predicted NLSs in the PRV UL42 and UL30 sequences identified with the PSORT II software program. The single-letter amino acid code is used. The superscript numbers indicate the corresponding amino acid positions within the protein sequence. **(B)** Schematic representation of EGFP, EGFP–SV40NLS, and full-length EGFP–UL42 and their truncated mutant derivatives, which were used to identify the putative NLS in UL42. The localization of various EGFP-expressing fusion proteins was categorized into five different patterns: N, exclusively nuclear; N > C, more nuclear than cytoplasmic; N = C, diffuse; C > N, more cytoplasmic than nuclear; and C, strictly cytoplasmic. The localization results are summarized on the right. **(C)** For each construct, 100 EGFP-expressing cells were scored from independent transfections in three repeated experiments and the relative percentages of the different subcellular localization categories of the fusion constructs were calculated. N, exclusively nuclear; N > C, more nuclear than cytoplasmic; N = C, diffuse; C > N, more cytoplasmic than nuclear; C, strictly cytoplasmic. **(D)** Representative localization images of various UL42–EGFP fusion protein constructs. Localization of the fusion proteins was analyzed as the fluorescent EGFP signal with confocal microscopy, and the DNA was stained with Hoechst reagent. The merged GFP and DAPI signals are shown. The image for each construct is representative of three independent transfection experiments. **(E)** Schematic representation of the classical SV40 TAg-NLS and the predicted UL42 pat4, pat7, or bipartite NLS fused between EGFP and β-Gal. “None” indicates that no specific motif was fused between these two reporter proteins. The localization of these constructs is summarized on the right. N, exclusively nuclear; C > N, more cytoplasmic than nuclear; C, strictly cytoplasmic. **(F)** For each construct, 100 EGFP-expressing cells were scored after independent transfections in three repeated experiments and the relative percentages of the different subcellular localizations of the fusion constructs were estimated. N, exclusively nuclear; C > N, more cytoplasmic than nuclear; C, strictly cytoplasmic. **(G)** Representative localization images of various EGFP–β-Gal fusion proteins containing the specific NLS. Localization of the fusion proteins was analyzed as the fluorescent EGFP signal using confocal microscopy, and the DNA was stained with Hoechst reagent. The merged GFP and DAPI signals are shown. The image for each construct is representative of three independent transfection experiments.

The results described above clearly suggest that nuclear transport of UL30 is dependent on UL42. Therefore, we hypothesized that UL42 contains an NLS motif that mediates the nuclear import of the DNA polymerase holoenzyme. A sequence analysis of UL42 and UL30 with the PSORT II program (Nakai and Horton, 1999) predicted that UL42 contains two putative monopartite NLS motifs at amino acids 367–370 (pat4) and amino acids 364–370 (pat7), and a bipartite NLS at amino acids 354–370, whereas UL30 has only one putative monopartite NLS at amino acids 537–543 (pat7) (Figure 2A). Because UL30 has a predicted molecular mass of approximately 116 kDa, which far exceeds the exclusion limit for passive diffusion through the NPC, we tested whether its predicted NLS (^537^PDKRRDI^543^) is responsible for the weak nuclear localization. However, the substitution of ^539^KRR^541^ with three A residues did not alter the localization of UL30 (data not shown), indicating that this putative NLS does not function as a real NLS. We speculated that the weak nuclear staining of UL30 in the transfected cells was associated with its single-stranded-DNA-binding activity. A small quantity of UL30 may bind to mitotic chromosomes after the breakdown of the nuclear envelope and is thus translocated into the nucleus. To verify the functionality of the three predicted NLS motifs in UL42, various fragments of UL42 containing the putative NLSs were fused to the reporter protein EGFP (Figure 2B), and the subcellular localization of the various EGFP fusion constructs was analyzed using confocal microscopy. Importantly, EGFP alone and some of the EGFP fusion proteins were small enough (less than 60–70 kDa) to passively diffuse between the nucleus and the cytoplasm. Taking this into account, we established a positive control construct expressing EGFP fused to a well-characterized SV40 TAg-NLS, which displayed strong nuclear localization, and a negative control construct expressing EGFP alone, which diffused throughout the cells. We compared the localization of various EGFP–UL42 fusion constructs with that of the positive and negative controls, and five different patterns were distinguished: exclusively nuclear (N), more nuclear than cytoplasmic (N > C), diffuse (N = C), more cytoplasmic than nuclear (C > N), and strictly cytoplasmic (C). The relative proportions of the five localization categories for each construct were scored in 100 EGFP-expressing cells in an independent transfection experiment.

The relative proportions are shown in Figure 2C, and the representative images of three independent localization experiments are shown in Figure 2D. EGFP–UL42(2–384), expressing the full-length UL42 fused to EGFP, was located exclusively in the nucleus of almost all cells (Figure 2C; N, 100%). By contrast, EGFP expressed alone produced a diffuse pattern (Figure 2C; N = C, > 95%), whereas EGFP fused to SV40 TAg-NLS mainly accumulated in the nucleus (Figure 2C; N, > 85%). EGFP–UL42(2–366) displayed strong cytoplasmic localization (Figure 2C; C, > 90%), whereas EGFP–UL42(367–384), containing the predicted pat4 NLS, showed a diffuse pattern, similar to that of wild-type EGFP (Figure 2C; N = C, > 85%). This indicates that the putative pat4 NLS is not the determinant that targets UL42 to the nucleus. EGFP–UL42(2–363) displayed predominantly cytoplasmic accumulation (Figure 2C; C > N, > 90%), whereas EGFP–UL42(364–384), containing the predicted pat7 NLS, diffused throughout the cells, similar to the localization of EGFP alone (Figure 2C; N = C, > 80%), suggesting that the putative pat7 NLS does not function as a real NLS. EGFP–UL42(2–370) and EGFP–UL42(354–384), containing the predicated bipartite NLS, localized strongly to the nucleus, similar to the positive construct EGFP–SV40NLS, with relative proportions of 100% (Figure 2C; N) and approximately 85% (Figure 2C; N), respectively. However, EGFP–UL42(371–384) showed diffuse localization, like that of wild-type EGFP (Figure 2C; N = C, > 95%), and EGFP–UL42(2–353) mainly accumulated in the cytoplasm (Figure 2C; C > N, about 70%; C, about 30%). These data imply that the putative bipartite NLS is responsible for the nuclear localization of UL42.

To further confirm the functionality of the three predicted NLS motifs, we examined whether they were sufficient to transport a heterologous protein into the nucleus. We chose β-Gal as the test protein because it is exclusively expressed in the cytoplasm because it is large and lacks an NLS motif. The relative proportions of various EGFP-β-Gal fusion proteins localization categories are shown in Figure 2F, and the representative images of three independent localization experiments are shown in Figure 2G. β-Gal localized exclusively in the cytoplasm (Figure 2F; C, 100%) when fused to the carboxy-terminus of EGFP (Figure 2E). Since the NLS of SV40 TAg is a canonical example of a functional and transferable NLS motif, we fused this NLS between EGFP and β-Gal to construct a positive control (Figure 2E). As expected, the positive construct displayed an exclusive nuclear fluorescent signal (Figure 2F; N, 100%). When the predicted UL42 pat4 and pat7 NLS motifs were fused between EGFP and β-Gal (Figure 2E), respectively, the fusion constructs EGFP–Pat4–β-Gal and EGFP–Pat7–β-Gal predominantly accumulated in the cytoplasm (Figure 2F; C > N, about 90%). When the predicted UL42 bipartite NLS was fused between EGFP and β-Gal (Figure 2E), the fusion construct EGFP–Bipartite–β-Gal was exclusively expressed in the nucleus (Figure 2F; N, 100%), indicating that this NLS translocated EGFP–β-Gal into the nucleus, and that it is a functional and transferable NLS motif. Collectively, these results suggest that the C-terminal peptide ^354^KRPAAPRMYTPIAKRPR^370^ functions as a classical bipartite NLS in UL42.

### K^354^, R^355^, and K^367^ are important for the function of the UL42 bipartite NLS

**Figure 3:**
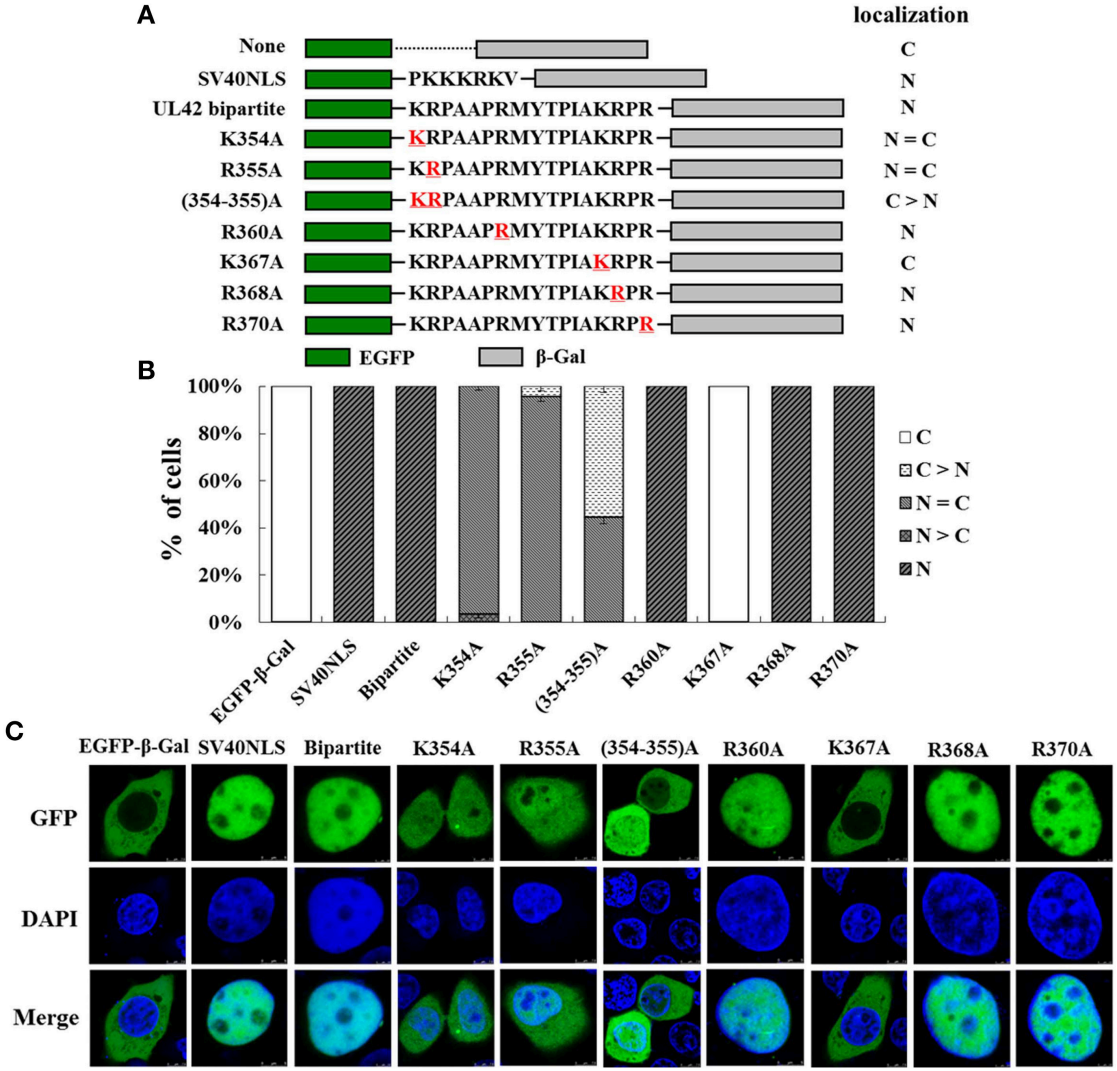
**K^354^, R^355^, and K^367^ are important for the function of the UL42 bipartite NLS. (A)** Schematic representation of the classical SV40 TAg-NLS, the UL42 bipartite NLS, and the UL42 NLS bearing amino acid substitutions fused between EGFP and β-Gal. “None” indicates that no specific motif was fused between these two reporter proteins. The red underlined amino acids were changed to A residue at the indicated positions. The localization of these constructs is summarized on the right. N, exclusively nuclear; N = C, diffuse; C > N, more cytoplasmic than nuclear; C, strictly cytoplasmic. **(B)** For each construct, 100 EGFP-expressing cells were scored after independent transfections in three repeated experiments and the relative percentages of the different subcellular localizations of the fusion constructs were estimated. N, exclusively nuclear; N > C, more nuclear than cytoplasmic; N = C, diffuse; C > N, more cytoplasmic than nuclear; C, strictly cytoplasmic. **(C)** Subcellular localization of various EGFP–β-Gal fusion proteins containing the specific NLS was analyzed with confocal microscopic analysis of the fluorescent EGFP signal and the Hoechst-reagent-stained DNA. The merged GFP and DAPI signals are shown. The image for each construct is representative of three independent transfection experiments.

To analyze in detail the contribution of the single basic amino acid residue to the function of the UL42 bipartite NLS, we constructed a series of mutant derivatives affecting the bipartite NLS in the context of EGFP–Bipartite–β-Gal (Figure 3A). EGFP–β-Gal and EGFP–SV40NLS–β-Gal were used as negative and positive controls, respectively. The relative proportions of various EGFP–β-Gal fusion proteins localization categories are shown in Figure 3B, and the representative images of three independent localization experiments are shown in Figure 3C. EGFP–β-Gal localized exclusively in the cytoplasm (Figure 3B; C, 100%), and EGFP–SV40NLS–β-Gal displayed an exclusive nuclear fluorescent signal (Figure 3B; N, 100%). When the UL42 bipartite NLS bearing point mutation R360A, R368A, or R370A was fused between EGFP and β-Gal, respectively, the fusion proteins were exclusively located at the nucleus (Figure 3B; N, 100%), resembling the localization of EGFP–Bipartite–β-Gal. When K^354^ and R^355^ were individually or both changed to A residues, the fusions EGFP–K354A–β-Gal and EGFP–R355A–β-Gal exhibited a diffuse pattern (Figure 3B; N = C, about 95%), and the fusion EGFP–(354–355)A–β-Gal mainly localized to the cytoplasm but produced partial nuclear fluorescent signal (Figure 3B; C > N, about 55%; N = C, about 45%). Intriguingly, when point mutation K367A was introduced into the UL42 bipartite NLS, the mutated NLS failed to transport the EGFP–β-Gal fusion protein into the nucleus (Figure 3B; C, 100%). Taken together, these results suggest that K^354^, R^355^, and K^367^ are important for the integral structure and function of the UL42 bipartite NLS.

### UL42 binds to importins α3 and α4 via its nls but not to importin α1, α5, α6, α7, α8, or β

**Figure 4:**
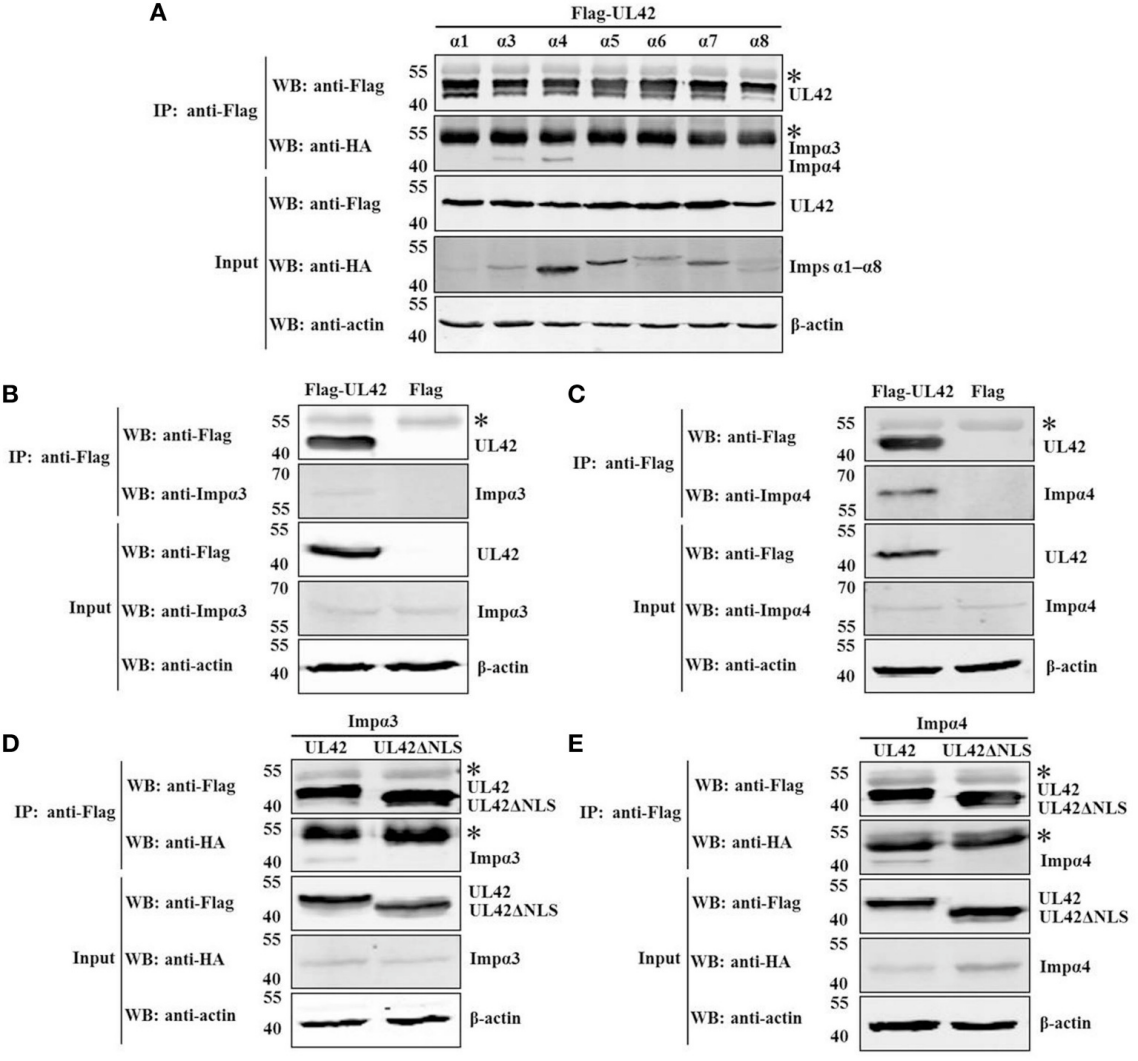
**UL42 binds to importins α3 and α4 through its NLS. (A)** Co-IP of HEK293T cells cotransfected with recombinant constructs encoding Flag–UL42 and HA-tagged importin α1, α3, α4, α5, α6, α7, or α8. **(B)** Co-IP of pCMV-Flag–UL42-transfected and untransfected HEK293T cells to identify the association between UL42 and endogenous importin α3. **(C)** Co-IP of pCMV-Flag–UL42-transfected and untransfected HEK293T cells to identify the association between UL42 and endogenous importin α4. **(D)** Co-IP of HEK293T cells cotransfected with recombinant constructs encoding Flag–UL42 or Flag–UL42ΔNLS and HA-tagged importin α3. **(E)** Co-IP of HEK293T cells cotransfected with recombinant constructs encoding Flag–UL42 or Flag–UL42ΔNLS and HA-tagged importin α4. IP was performed using an MAb recognizing the Flag tag, and the western blotting (WB) was probed with the antibodies indicated on the left. The cytoskeletal protein β-actin was used as the internal control. The asterisk indicates the heavy chain of IgG. The positions of the molecular mass markers (kDa) are indicated on the left. The WB results are representative of three or more independent experiments.

The results described above demonstrate that UL42 contains a classical bipartite NLS to direct it to the nucleus. It has been shown that bipartite NLS motifs typically interact with importin α and require the formation of the NLS-containing cargo–importin α/β heterotrimer for their nuclear import (Marfori et al., 2011, 2012). Therefore, we hypothesized that the nuclear import of UL42 is probably mediated by the importin α/β pathway. To date, seven different isoforms of importin α have been characterized in mammalian cells (Hogarth et al., 2006; Tejomurtula et al., 2009; Kelley et al., 2010), but no information about the binding activity of the different importin α proteins to UL42 has been reported. To verify our hypothesis, we tested the formation of the UL42–importin α complex in cotransfected HEK293T cells using co-IP assays of Flag-tagged UL42 and various HA-tagged truncated importin α proteins that lacked the autoinhibitory IBB domain and thus bound to the NLS with an affinity similar to that of the importin α/β heterodimer (Kobe, 1999; Fontes et al., 2003b). HEK293T cells were cotransfected with constructs expressing Flag–UL42 and HA-tagged importin α1, α3, α4, α5, α6, α7, or α8, and IP was performed on the cotransfected cell lysates with an anti-Flag MAb. As shown in Figure 4A, HA-tagged importins α3 and α4 were coimmunoprecipitated with Flag–UL42. When IP was performed with an anti-HA MAb, Flag–UL42 was only detected in the proteins coimmunoprecipitated with HA-tagged importins α3 and α4 (data not shown). These data confirm that UL42 interacts with importins α3 and α4. We also investigated whether UL42 interacts with endogenous importins α3 and α4. IP was performed on lysates from pCMV-Flag–UL42-transfected or untransfected HEK293T cells with an MAb that recognized Flag. Endogenous importins α3 and α4 were detected in the proteins immunoprecipitated from the lysates of pCMV-Flag-UL42-transfected cells but not in those in the lysates of untransfected cells (Figures 4B,C), demonstrating an endogenous association between UL42 and importin α3 or α4. The NLS dependence of these interactions was confirmed with an NLS-null mutant, Flag–UL42ΔNLS, constructed based on the data above, which showed that the deletion of the bipartite NLS of UL42 abolished the nuclear import of full-length UL42 fused to EGFP. HA-tagged importins α3 and α4 were coimmunoprecipitated with Flag–UL42 but not with Flag–UL42ΔNLS, whether IP was performed with an anti-Flag (Figures 4D,E) or anti-HA MAb (data not shown), confirming that the deletion of this NLS abolished the binding of UL42 to importins α3 and α4, and that their interactions are undoubtedly mediated by this NLS motif.

The interaction between UL42 and importin α3 or α4 suggests that the nuclear import of UL42 is most likely to be mediated by the importin α/β pathway. It has been demonstrated that importin β alone is sufficient to transport some nuclear proteins into the nucleus, without the requirement of importin α (Palmeri and Malim, 1999; Singhal et al., 2006). This prompted us to investigate whether UL42 interacts with importin β. However, importin β was not coimmunoprecipitated with UL42 from the lysates of cells coexpressing HA-tagged importin β and Flag–UL42 (data not shown). When IP was performed on the lysates of pCMV-Flag–UL42-transfected or untransfected cells with an anti-Flag MAb, endogenous importin β was not detected in the proteins immunoprecipitated from the transfected cell lysates with an MAb recognizing importin β (data not shown). Thus, whether importin β is involved in the nuclear import of UL42 or not remains to be investigated.

### *In vitro* nuclear import of UL42 is a cytosol-, temperature-, and energy-dependent process and requires the NLS

**Figure 5:**
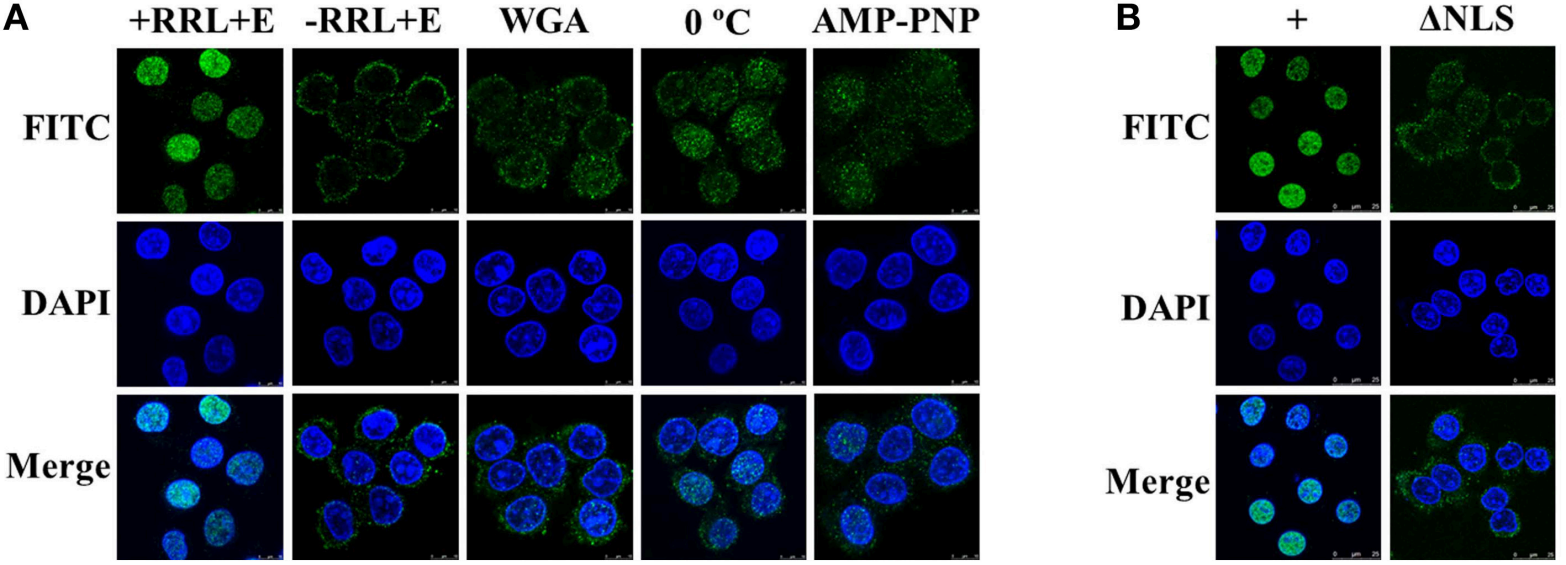
**Nuclear import of UL42 is an active process that depends on cytosolic factors and the NLS, and occurs in a temperature- and energy-dependent manner. (A)** After the permeabilization of the cell membranes with digitonin and the depletion of soluble cytosolic factors by consecutively washing the cells with ice-cold TB, HeLa cells were incubated with an import mixture containing rabbit reticulocyte lysate (RRL), an ATP-regenerating system, and an import substrate of purified Flag–UL42 in TB (+RRL+E). –RRL+E, RRL was omitted from the import mixture. WGA, the cells were pretreated with WGA before incubation with the import mixture. 0°C, the nuclear import assay was performed on ice. AMP–PNP, the ATP-regenerating system was omitted and ATP was replaced with 1 mM AMP–PNP. The nuclear uptake was analyzed with immunofluorescence assays using an MAb recognizing the Flag tag. The merged FITC and DAPI signals are shown. Images are representative of three independent nuclear import assays. **(B)** +, equivalent to “+RRL+E” in **(A)**. ΔNLS, the import substrate (purified Flag–UL42) was omitted and the purified Flag–UL42ΔNLS was added. The merged FITC and DAPI signals are shown. Images are representative of three independent nuclear import assays.

To characterize the nuclear import pathway of UL42 in more detail, *in vitro* nuclear import assays using digitonin-permeabilized HeLa cells were performed. For this assay, the cytoplasmic membranes of the HeLa cells were first permeabilized with digitonin and the soluble cytosolic factors were washed out. The nuclear import of Flag–UL42 was then investigated in the presence of rabbit reticulocyte lysate (RRL) as a complete source of exogenous nuclear import factors. After the addition of an ATP-regenerating system, Flag–UL42 was readily imported into the nuclei of the permeabilized cells (Figure 5A, +RRL+E). By contrast, when RRL was omitted from the import mixture, nuclear import was abolished and the fluorescent signals were confined to the cytoplasm (Figure 5A, –RRL+E), indicating that soluble cytosolic factors are essential for the nuclear import of UL42. Wheat-germ agglutinin (WGA) is a lectin that binds to N-acetylglucosamine-modified nucleoporins and thus inhibits some nuclear transport pathways, but does not affect the passive diffusion through the NPC (Kobe, 1999; Fontes et al., 2003b). To confirm the specificity of this nuclear import mechanism, WGA was preincubated with the permeabilized cells before the import mixture was added. Nuclear import was massively inhibited (Figure 5A, WGA), indicating that this import mechanism is specific. Furthermore, the nuclear import was inhibited when the import mixture was incubated on ice (Figure 5A, 0°C) or when the ATP-regenerating system was omitted and ATP was replaced with nonhydrolyzable AMP–PNP (Figure 5A, AMP–PNP). This suggests that the nuclear importation of UL42 is a temperature- and energy-dependent process. No nuclear import was detected when the import substrate, purified Flag–UL42, was replaced with Flag–UL42ΔNLS lacking the bipartite NLS (Figure 5B, ΔNLS), demonstrating that the UL42 NLS is essential for the nuclear accumulation of UL42 in an *in vitro* system and that the import in digitonin-permeabilized cells and in living cells occurs *via* functionally similar import mechanisms. Taken together, these findings show that UL42 is transported into the nuclei of digitonin-permeabilized HeLa cells in a cytosol-, temperature-, energy-, and NLS-dependent manner.

### Nuclear import of UL42 is mediated by the importin α/β pathway

**Figure 6:**
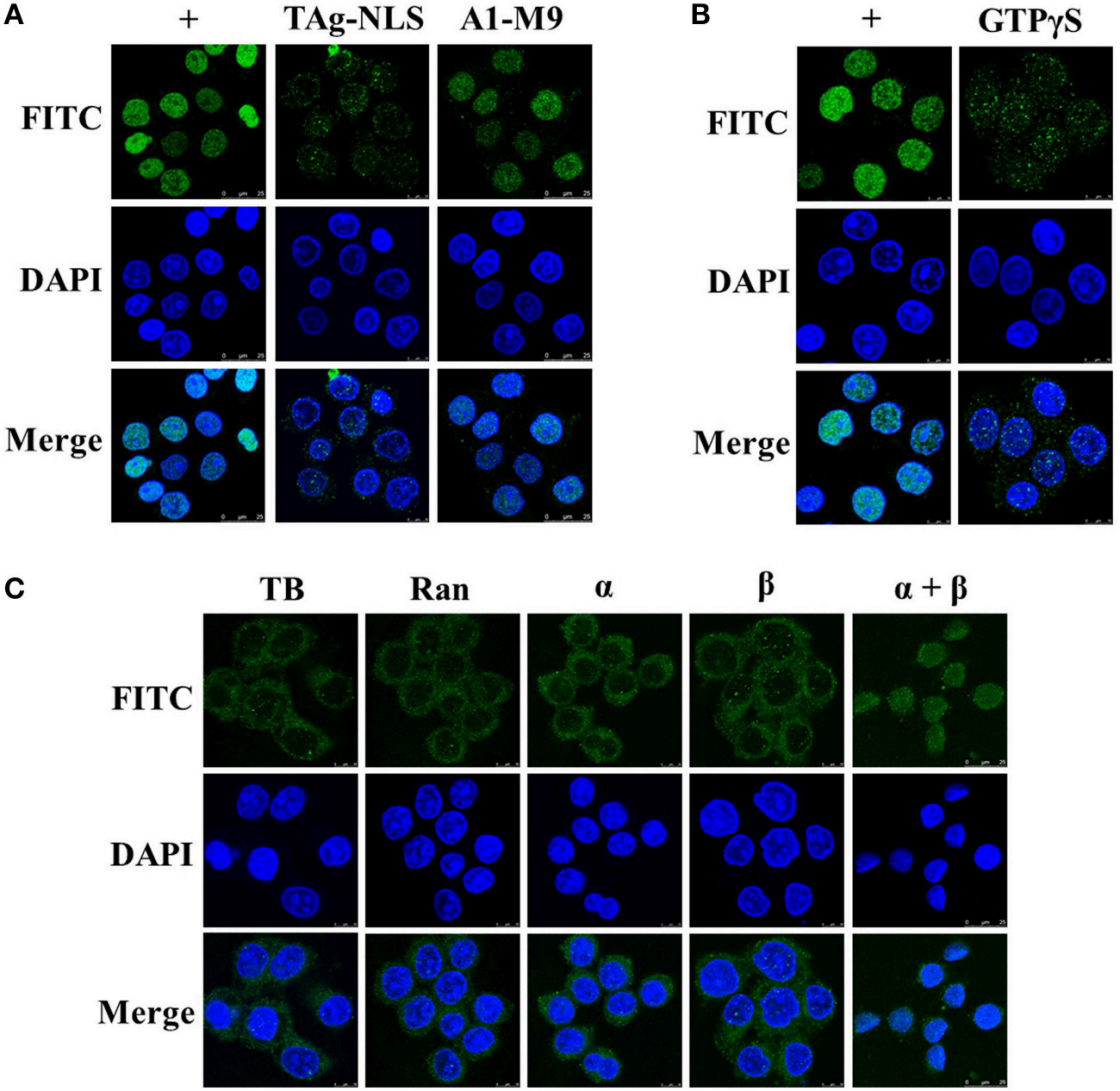
**UL42 is transported into the nucleus *via* the importin α/β pathway. (A)** UL42 competes with the SV40 TAg–NLS, but not with the hnRNP A1-M9, for nuclear import. Nuclear import was performed in the presence of RRL, an ATP-regenerating system, and purified Flag–UL42 (+). TAg–NLS, 1 mM SV40 TAg–NLS peptide was added to the import mixture. A1-M9, 1 mM hnRNP A1-M9 peptide was added to the import mixture. The merged FITC and DAPI signals are shown. Images are representative of three independent nuclear import assays. **(B)** GTP dependence of UL42 nuclear uptake. Nuclear import was performed in the presence of RRL, an ATP-regenerating system, and purified Flag–UL42 (+). GTPγS, GTP was omitted and 1 mM GTPγS was added. The merged FITC and DAPI signals are shown. Images are representative of three independent nuclear import assays. **(C)** UL42 gains entry to the nucleus in the presence of importins α and β. Reconstitution assays were performed in transport buffer (TB), with a Ran mixture (3 μM Ran and 0.5 μM NTF2) (Ran), and the addition of 1 μM purified importin α4 (α) or 1 μM importin β (β) or 1 μM concentrations of both importin α4 and importin β (α+β), with 200 μg/ml purified Flag–UL42 as the import substrate. The merged FITC and DAPI signals are shown. Images are representative of three independent nuclear import assays.

To clarify whether importins α and β are both functionally involved in the nuclear importation of UL42 in digitonin-permeabilized HeLa cells, we determined whether a well-recognized competitor peptide that targets specific nuclear import receptors competitively inhibited the nuclear import process. SV40 TAg requires both importins α and β to target it to the nucleus, and its NLS has been successfully used to investigate the nuclear import pathways of several nuclear proteins in competition experiments (Subramaniam et al., 1999; Lischka et al., 2003). Therefore, we chose SV40 TAg-NLS as the inhibitory peptide, which has been shown to directly bind to various importin α isoforms (Sekimoto et al., 1997; Bian et al., 2007). We selected the hnRNP A1-M9 peptide as the negative control, which binds to the nuclear transport receptor transportin (importin β2) and therefore does not interfere with the classical importin α/β-mediated nuclear import pathway (Pollard et al., 1996; Bonifaci et al., 1997). As shown in Figure 6A, the import substrate Flag–UL42 was imported into the nuclei of digitonin-permeabilized HeLa cells when no competitor peptide was present in the import mixture (Figure 6A, +). However, this nuclear import was inhibited after the addition of an excess of the SV40 TAg-NLS peptide (Figure 6A, TAg-NLS). By contrast, an excess of the A1-M9 peptide did not affect the nuclear import process (Figure 6A, A1-M9), indicating that the inhibition caused by the SV40 TAg-NLS peptide was specific. These results suggest that UL42 competes with the SV40 TAg-NLS peptide, but not with the hnRNP A1-M9 peptide, for binding to importin α and nuclear import. However, we could not infer from the competition experiments that importin β participates in this nuclear import process because the SV40 TAg-NLS peptide only disrupts the binding of UL42 to importin α. In the classical importin α/β pathway, importin β only binds to the cargo *via* the adaptor molecule importin α in the cytoplasm after its dissociation from RanGTP by GTP hydrolysis, and it releases the cargo in the nucleus after it binds to RanGTP (Lange et al., 2007; Marfori et al., 2011). Therefore, this pathway is inhibited by GTPγS, a nonhydrolyzable analog of GTP (Melchior et al., 1993; Moore and Blobel, 1993). As shown in Figure 6B, nuclear import was inhibited when GTP was replaced with GTPγS, suggesting that importin β may be involved in the nuclear uptake of UL42. Taken together, these results convincingly indicate that the importin α/β heterodimer is required for the nuclear accumulation of UL42.

Nuclear proteins containing classical NLS motifs can gain access to the nucleus only through the importin-β-mediated pathway, without the need for the adaptor molecule importin α (Palmeri and Malim, 1999; Singhal et al., 2006). Therefore, the data from the competition and inhibition assays do not exclude the possibility that importin β alone is sufficient to mediate the nuclear import of UL42. To answer this, we performed a reconstitution assay in which RRL was replaced with Ran and importins α and β. As shown in Figure 6C, Ran alone or Ran in association with importin α or β was insufficient to transport UL42 into the nucleus. It was only with the combination of Ran and importins α and β that the nuclear accumulation of UL42 was detected, confirming the requirement for the importin α/β heterodimer. Collectively, the nuclear localization of UL42 is mediated by the importin α/β nuclear import pathway.

### UL42 NLS is essential for the nuclear import of the PRV DNA polymerase holoenzyme

**Figure 7:**
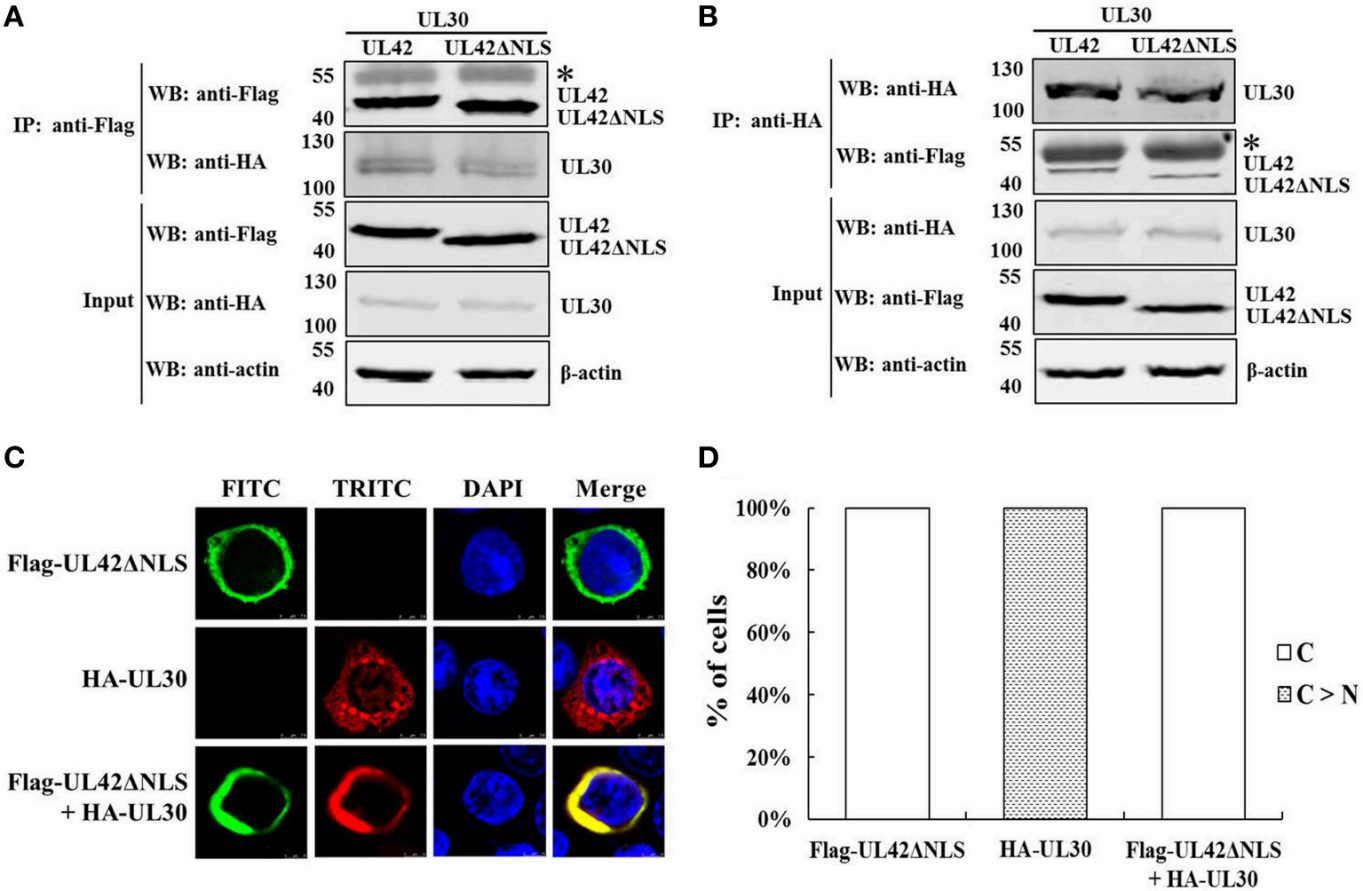
**An UL42 NLS-null mutant confined the PRV DNA polymerase holoenzyme to the cytoplasm. (A)** Co-IP of HEK293T cells transfected with pCMV-Flag–UL42ΔNLS and/or pCAGGS-HA–UL30. IP was performed with an MAb recognizing the Flag tag, and the western blotting (WB) was probed with the antibodies indicated on the left. The cytoskeletal protein β-actin was used as the internal control. The asterisk indicates the heavy chain of IgG. The positions of the molecular mass markers (kDa) are indicated on the left. The WB results are representative of three or more independent experiments. **(B)** Reverse Co-IP of HEK293T cells transfected with pCMV-Flag–UL42ΔNLS and/or pCAGGS-HA–UL30. IP was performed with an MAb recognizing the HA tag, and the WB was probed using the antibodies indicated on the left. The cytoskeletal protein β-actin was used as the internal control. The asterisk indicates the heavy chain of IgG. The positions of the molecular mass markers (kDa) are indicated on the left. The WB results are representative of three or more independent experiments. **(C)** HeLa cells transfected with pCMV-Flag–UL42ΔNLS and/or pCAGGS-HA–UL30 were analyzed by immunofluorescence assays using an MAb recognizing the Flag tag (FITC, green) and/or a PcAb recognizing the HA tag (TRITC, red). The DNA was stained with Hoechst reagent (DAPI, blue). The merged FITC, TRITC, and DAPI signals are shown. Image for each construct is representative of three independent transfection experiments. **(D)** To analyze the localization patterns of Flag–UL42ΔNLS and HA–UL30 statistically, 100 positive cells expressing Flag–UL42ΔNLS or HA–UL30 or coexpressing Flag–UL42ΔNLS and HA–UL30 were scored from independent transfections in three repeated experiments and the relative percentages of the different subcellular localization categories were calculated. C > N, more cytoplasmic than nuclear; C, strictly cytoplasmic.

The previous data confirmed that UL42 can transport UL30 into the nucleus after their assembly into a heterodimer in the cytoplasm (Figures 1C,D). To investigate whether the UL42 NLS influences the localization of the PRV DNA polymerase holoenzyme, the colocalization of HA–UL30 and an UL42 NLS-null mutant, Flag–UL42ΔNLS, was examined. Although the NLS had been deleted, Flag–UL42ΔNLS could also physically interact with HA–UL30 (Figures 7A,B). Expectedly, unlike wild-type UL42 (Figures 1C,D), Flag–UL42ΔNLS was only detected in the cytoplasm when transfected alone because of the lack of the NLS (Figures 7C,D). When HA–UL30 and Flag–UL42ΔNLS were coexpressed, the UL42/UL30 heterodimer was completely confined to the cytoplasm (Figures 7C,D), indicating that UL30 utilizes the NLS function of UL42 for its translocation into the nucleus. These results suggest that the UL42 NLS is essential for transporting the PRV DNA polymerase holoenzyme into the nucleus.

## Discussion

The nuclear targeting of the PRV DNA polymerase holoenzyme is a prerequisite for its function in the initiation of viral DNA replication. This study is the first to investigate the nuclear transport mechanisms of the PRV DNA polymerase catalytic and accessory subunits. It is demonstrated that nuclear transport of the PRV DNA polymerase catalytic subunit UL30 is dependent on the accessory subunit UL42 in an *in vitro* expression system, whereas UL42 is transported into the nucleus through the classical importin α/β-mediated nuclear import pathway.

It has been shown that the DNA polymerase processivity factors of several herpesviruses, including UL42 of HSV-1 (Alvisi et al., 2008), UL44 of HCMV (Alvisi et al., 2005), U27 of human herpesvirus 7 (HHV-7) (Takeda et al., 2000), BMRF1 of EBV (Zhang et al., 1999), and PF-8 of KSHV (Chen et al., 2005), independently localize to the nucleus and contain a functional NLS. Like these processivity factors, we confirmed that the PRV DNA polymerase processivity factor UL42 also localizes to the nucleus when expressed alone (Figures 1C,D) and contains a functional bipartite NLS (Figure 2). Remarkably, a single amino acid substitution, K367A, was sufficient to abolish the capacity of the UL42 bipartite NLS to transport EGFP–β-Gal into the nucleus, indicating that K^367^ is essential for the function of the UL42 bipartite NLS. This is consistent with the previous report that the K residue [highlighted in bold in the consensus sequence of a bipartite NLS KR(X)_10−12_**K**(K/R)X(K/R)] is the most critical determinant of the NLS structure and function (Fontes et al., 2003a; Kosugi et al., 2009; Marfori et al., 2012). With respect to the NLS categories, the processivity factors of HCMV, HHV-7, EBV, and KSHV have been shown to be monopartite, closely analogous to that identified on SV40 TAg (Zhang et al., 1999; Takeda et al., 2000; Alvisi et al., 2005; Chen et al., 2005). In the case of HSV-1 and PRV processivity factors, however, their NLSs have been demonstrated to be bipartite, with two clusters of basic amino acids being necessary for optimal nuclear localization (Alvisi et al., 2008). Comparison of the identified NLSs and several predicted NLSs of the processivity factors among the α, β, and γ herpesviruses indicates that the NLSs in the α herpesviruses (HSV-1, HSV-2, and PRV) appear to be conserved and bipartite, and the β herpesviruses (HCMV, HHV-6, and HHV-7) seem to have conserved monopartite NLS motifs, whereas the γ herpesviruses (EBV and KSHV) have divergent monopartite NLS motifs (data not shown). Thus, the NLS differs among different herpesviruses. Interestingly, what these NLSs have in common is that they are all located at the carboxy-terminus. However, it remains unclear how and why HSV-1 and PRV have evolved a bipartite NLS on their processivity factors due to the limitations of current knowledge on herpesvirus biology, but this may imply that they have additional functions during the viral life cycle, such as infecting neuronal cells (Alvisi et al., 2013).

In this study, we demonstrated that UL42 is imported into the nucleus through the importin α/β pathway, in which importins α3 and α4, as the adaptor molecules, bridge UL42 and importin β, whereas importin β mediates the interaction between the importin α/β–UL42 heterotrimer and the NPC. Interestingly, HSV-1 UL42 and HCMV UL44 have also been shown to gain entry to the nucleus by the importin α/β heterodimer (Alvisi et al., 2005, 2008). Importins α3 and α4 belong to the α-Q subfamily (Hogarth et al., 2006), thus UL42 has unique specificity and affinity for the members of this subfamily. By contrast, HSV-1 UL42 has specificity and affinity for both α-Q and α-S subfamily members, while HCMV UL44 only binds efficiently to α-S subfamily members (Alvisi et al., 2008). Although the nuclear transport receptors responsible for nuclear localization of the EBV and KSHV processivity factors have not been identified experimentally, the transport is probably mediated by the importin α/β pathway, since they have highly basic NLS motifs resembling the SV40 TAg-NLS (Alvisi et al., 2013).

Surprisingly, not all known herpesvirus DNA polymerase catalytic subunits are able to localize independently to the nucleus. Among all identified herpesvirus DNA polymerase catalytic subunits, only HSV-1 and HCMV catalytic subunits are capable of targeting to the nucleus when expressed alone (Alvisi et al., 2006, 2007). HSV-1 DNA polymerase catalytic subunit UL30 has been shown to possess an importin α/β-recognized NLS to direct its nuclear localization, even in the absence of the processivity factor (Alvisi et al., 2007, 2008). Also, HCMV DNA polymerase catalytic subunit UL54 has a functional NLS to mediate its nuclear import *via* the importin α/β heterodimer (Alvisi et al., 2006). In the case of EBV and KSHV catalytic subunits, the NLS appears to be absent and the nuclear localization is completely dependent on the coexpression with their processivity factors *via* a piggy-back mechanism (Chen et al., 2005; Kawashima et al., 2013). Like EBV and KSHV catalytic subunits, PRV UL30 was demonstrated to have no true NLS and the transport entirely relies on its coexpression with UL42 (Figures 1C,D). Remarkably, it was demonstrated that nuclear transport of HSV-1 UL30 and EBV BALF5 required molecular chaperone Hsp90 (Burch and Weller, 2005; Kawashima et al., 2013); HSV-1 UL30 got stabilized by interaction with Hsp90 for proper localization to the nucleus (Burch and Weller, 2005), and Hsp90 promoted the association of EBV BALF5 with BMRF1 for optimal nuclear localization of BALF5 (Kawashima et al., 2013). However, PRV UL30 appeared to do not require Hsp90 for nuclear import, since the nuclear localization of UL30 upon coexpression with UL42 was not affected by Hsp90 inhibitor radicicol (data not shown). It is not clear why Hsp90 is involved in the nuclear transport of HSV-1 UL30 and EBV BALF5, but not in the case of PRV UL30; this may reflect the differences of different host factors in the regulation of herpesvirus DNA replication. Therefore, nuclear transport mechanisms of the DNA polymerase catalytic subunits differ among herpesviruses.

In an UL42 NLS-null mutant, we confirmed that the deletion of the bipartite NLS in UL42 completely confined the UL42/UL30 heterodimer in the cytoplasm (Figures 7C,D), strongly suggesting that the function of this NLS is crucial for the nuclear targeting of the PRV DNA polymerase holoenzyme. It seems likely that the PRV DNA polymerase holoenzyme is transported into the nucleus only as an UL42/UL30 heterodimer complex after its assembly in the cytoplasm. This transport mechanism is analogous to what reported in the case of EBV and KSHV DNA polymerase holoenzymes (Chen et al., 2005; Kawashima et al., 2013), and may provide a means for the coordination and facilitation of the processes of viral DNA recognition and processive DNA synthesis (Alvisi et al., 2013). But unlike what reported in the case of HSV-1 and HCMV DNA polymerase holoenzymes, wherein their catalytic and accessory subunits can be imported individually, or as fully assembled holoenzyme complexes (Alvisi et al., 2005, 2006, 2007, 2008). It remains unclear why different herpesviruses have evolved different mechanisms to ensure the appropriate nuclear targeting of their DNA polymerase holoenzymes. It is possible that the capacity of HSV-1 and HCMV DNA polymerase catalytic subunits to be independently transported into the nucleus is essential to their infectious cycles (Alvisi et al., 2013). However, the inability of PRV, EBV, and KSHV DNA polymerase catalytic subunits to be independently transported into the nucleus is probably because their NLSs have been inactivated by mutagenesis during the course of virus evolution. Taken together, these results indicate that in addition to increasing the processivity of UL30 on the viral DNA, UL42 also functions as the nuclear cotransporter of UL30. Also, the bipartite NLS in UL42 may play a pivotal role in PRV replication by stabilizing the nuclear targeting of the DNA polymerase holoenzyme. Future studies are certainly needed to elucidate the specific role of the bipartite NLS in UL42 in PRV DNA replication. It is clear that this bipartite NLS is a promising target in therapeutic strategies to inhibit PRV replication by abrogating nuclear localization of the DNA polymerase holoenzyme. These intriguing possibilities will be the focus of future work in our laboratory.

**Figure 8:**
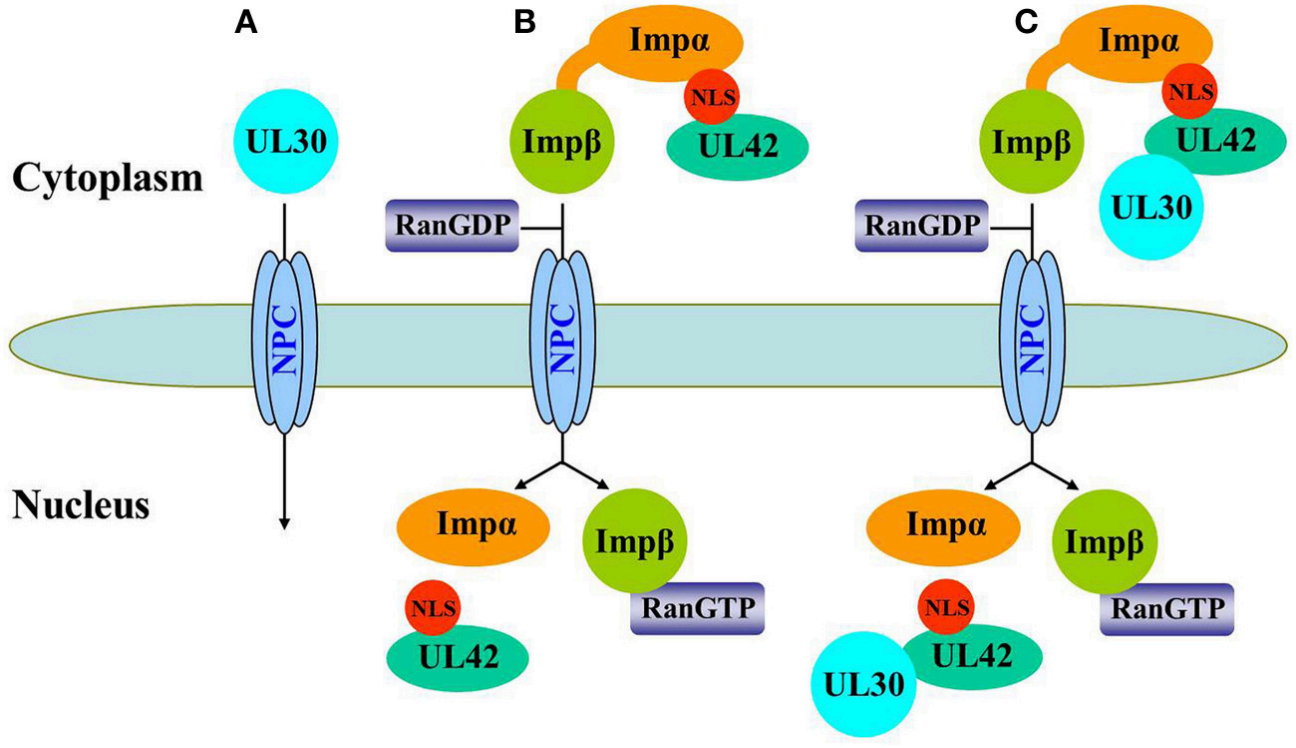
**Schematic representation of the nuclear import pathway for the PRV DNA polymerase holoenzyme. (A)** UL30 is not independently transported into the nucleus because it is large (116 kDa) and lacks a functional NLS. **(B)** UL42 can be independently imported into the nucleus *via* the classical importin α/β pathway. First, UL42 binds to the importin α/β heterodimer *via* its bipartite NLS to form a heterotrimeric complex in the cytoplasm, which then binds to the NPC *via* importin β. Second, the complex is translocated into the nucleus, where UL42 is released from the complex when importin β binds to nuclear RanGTP, and the transport receptors importins α and β are then recycled back to the cytoplasm for another round of import. **(C)** Nuclear import of the PRV DNA polymerase holoenzyme relies on the bipartite NLS present in its accessory subunit UL42. The UL42/UL30 holoenzyme complex was first assembled in the cytoplasm and then transported into the nucleus by the importin α/β pathway.

In conclusion, we have demonstrated that UL42 contains a functional and transferable bipartite NLS that mediates its nuclear localization by the classical importin α/β pathway. The bipartite NLS in UL42 is not only essential for the import of free UL42 but also for the transport of the PRV DNA polymerase holoenzyme in an *in vitro* expression system. Figure 8 shows a model of the nuclear import pathway of the PRV DNA polymerase holoenzyme: A, the catalytic subunit UL30 is not transported independently into the nucleus without the help of other cotransporters; B, the accessory subunit UL42 is independently transported into the nucleus by the classical importin α/β-mediated nuclear import pathway; and C, the DNA polymerase holoenzyme is translocated into the nucleus only as an UL42/UL30 heterodimer complex after its assembly in the cytoplasm, with the processivity factor UL42 acting as a nuclear cotransporter. Whether other viral proteins or host factors are involved in the nuclear transport of PRV UL30 needs to be determined in the future. This study expands the diversity of the nuclear transport mechanisms of the DNA polymerase holoenzymes among herpesviruses and has great significance for the further understanding of PRV replication.

## Author contributions

YPW, LF, and CL conceived and designed the study. YPW conducted the bulk of the experiments, analyzed the results, and wrote the manuscript. WD constructed recombinant plasmids expressing various EGFP fusion proteins. LH, LF, and CL reviewed the results and critically revised the manuscript. YWW and HW provided technical assistance during the experiments. All authors approved the final version of the manuscript.

### Conflict of interest statement

The authors declare that the research was conducted in the absence of any commercial or financial relationships that could be construed as a potential conflict of interest.
